# Identification of genome-wide single nucleotide polymorphisms in allopolyploid crop *Brassica napus*

**DOI:** 10.1186/1471-2164-14-717

**Published:** 2013-10-20

**Authors:** Shunmou Huang, Linbin Deng, Mei Guan, Jiana Li, Kun Lu, Hanzhong Wang, Donghui Fu, Annaliese S Mason, Shengyi Liu, Wei Hua

**Affiliations:** Key Laboratory of Biology and Genetic Improvement of Oil Crops, Ministry of Agriculture, Oil Crops Research Institute of the Chinese Academy of Agricultural Sciences, Wuhan, 430062 Hubei People’s Republic of China; The Oilseed Crop Institute, Hunan Agricultural University, National Oilseed Crop Improvement Center, Changsha, 410128 Hunan People’s Republic of China; Chongqing Rapeseed Technology Research Center, Chongqing Key Laboratory of Crop Quality Improvement, Key Laboratory of Biotechnology and Crop Quality Improvement of Ministry of Agriculture, College of Agronomy and Biotechnology, Southwest University, 216 Tiansheng Road, Beibei, Chongqing 400716 People’s Republic of China; Key Laboratory of Crop Physiology, Ecology and Genetic Breeding, Ministry of Education, Agronomy College, Jiangxi Agricultural University, Nanchang, 330045 China; Centre for Integrative Legume Research and School of Agriculture and Food Sciences, The University of Queensland, Brisbane, 4072 Australia

**Keywords:** *Brassica napus*, Allopolyploid, Resequencing, Genotyping, GoldenGate, Non-synonymous SNP

## Abstract

**Background:**

Single nucleotide polymorphisms (SNPs) are the most common type of genetic variation. Identification of large numbers of SNPs is helpful for genetic diversity analysis, map-based cloning, genome-wide association analyses and marker-assisted breeding. Recently, identifying genome-wide SNPs in allopolyploid *Brassica napus* (rapeseed, canola) by resequencing many accessions has become feasible, due to the availability of reference genomes of *Brassica rapa* (2n = AA) and *Brassica oleracea* (2n = CC), which are the progenitor species of *B. napus* (2n = AACC). Although many SNPs in *B. napus* have been released, the objective in the present study was to produce a larger, more informative set of SNPs for large-scale and efficient genotypic screening. Hence, short-read genome sequencing was conducted on ten elite *B. napus* accessions for SNP discovery. A subset of these SNPs was randomly selected for sequence validation and for genotyping efficiency testing using the Illumina GoldenGate assay.

**Results:**

A total of 892,536 bi-allelic SNPs were discovered throughout the *B. napus* genome. A total of 36,458 putative amino acid variants were located in 13,552 protein-coding genes, which were predicted to have enriched binding and catalytic activity as a result. Using the GoldenGate genotyping platform, 94 of 96 SNPs sampled could effectively distinguish genotypes of 130 lines from two mapping populations, with an average call rate of 92%.

**Conclusions:**

Despite the polyploid nature of *B. napus*, nearly 900,000 simple SNPs were identified by whole genome resequencing. These SNPs were predicted to be effective in high-throughput genotyping assays (51% polymorphic SNPs, 92% average call rate using the GoldenGate assay, leading to an estimated >450 000 useful SNPs). Hence, the development of a much larger genotyping array of informative SNPs is feasible. SNPs identified in this study to cause non-synonymous amino acid substitutions can also be utilized to directly identify causal genes in association studies.

## Background

It is estimated that approximately 70% of angiosperms have experienced one or more chromosome doubling events during their evolutionary history [1]. Many crop species are also polyploid, including *Brassica napus* (rapeseed, canola), *Triticum aestivum* (wheat), *Solanum tuberosum* (potato), *Gossypium hirsutum* (cotton), *Avena sativa* (oat) and *Saccharum officinarum* (sugarcane). *Brassica napus* is an allopolyploid species originating from interspecific hybridization between diploid progenitors *Brassica oleracea* and *Brassica rapa*[2], which are themselves derived from ancient polyploidy events resulting in genome triplication 13 to 17 million years ago [3, 4]. These recent and ancient polyploidy events resulted in numerous duplicated segments and homoeologous regions within the genome of *B. napus*[5]. Hence, discriminating between a) two homologous sequences and b) two nearly-identical homoeologous sequences is complex and difficult in *B. napus*[6].

SNPs (single-nucleotide polymorphisms) are single-nucleotide substitutions of one base for another in DNA sequences. SNPs are quite abundant throughout the entire genome of most organisms and every SNP in low copy DNA is a potentially useful marker [7]. SNP markers have been applied in studies of genetic variation, construction of genetic maps, population structure analysis, association genetics, map-based gene isolation, and other plant breeding applications [8]. In contrast to traditional SNP detection techniques, detecting SNPs using next generation sequencing (NGS) technologies (such as Illumina sequencing, Roche 454 sequencing, Applied Biosystems SOLiD Sequencing and Helicos Biosciences Corporation Heliscope Sequencing) is high-throughput, low cost and high efficiency. Hence, next generation sequencing has been used in the development of thousands of molecular markers in many species [9–11], such as *Oryza sativa* (rice) [12, 13], *Helianthus annuus* (sunflower) [14], *Zea mays L*. (maize) [15], *Triticum aestivum* (wheat) [16], *Manihot esculenta* (cassava) [17] and *Arabidopsis thaliana* (thale cress) [18]. Mass SNP information has already been successfully used for genome-wide association studies [9, 19–21], and SNP markers are increasingly becoming the optimal marker system.

In recent years, many SNPs have been discovered in *B. napus*[22, 23] and *B. oleracea*[24]. However, these SNPs are inadequate for large-scale applications [23]. Trick et al. [23] used Solexa sequencing to generate approximately 20 million expressed sequence tags (ESTs) from two *B. napus* cultivars. They obtained 23,330-41,593 (two accessions) putative SNPs through alignment to a publicly available set of approximately 94,000 *Brassica* species unigenes [23]. However, 87.5-91.2% of the putative SNPS were ‘hemi-SNPs’, amplifying two or more different genomic loci. In comparison with ‘hemi-SNPs’, ‘simple SNPs’ are derived from allelic differences at a single genomic locus. Similarly, Bancroft et al. [25] used transcriptome sequencing to construct two *B. napus* linkage maps from 21,323 and 1,714 SNP markers, but discovered that the first map comprised 16,800 (78.8%) hemi-SNP types and only 4,124 (19.8%) simple SNP types, and that the second map comprised 1,266 hemi-SNPs and only 409 simple SNPs [25]. Later, Bus et al. [26] used eight different *B. napus* germplasm types to identify genome-wide restriction-site associated DNA (RAD) fragments, and obtained over 20,000 SNPs [26]. Hence, usability and availability of SNPs in *B. napus* is still limited, and development of a large set of simple SNP markers is highly desirable.

At present, both the A and C progenitor genomes (*B. rapa* and *B. oleracea*) of *B. napus* have been sequenced. The *B. rapa* A genome was released in 2011 [27], and the *B. oleracea* C genome has been sequenced by a collaboration between the OCRI (Oil Crops Research Institute of Chinese Academy of Agricultural Sciences) and other research communities and hence could also be used for BLAST analysis using the BRAD database [28] (http://brassicadb.org/brad/), although the C genome sequences have not yet been released. Based on these available reference sequences, large-scale identification of simple SNPs in *B. napus* could be implemented. The objective of the present study was to develop a set of genome-wide and evenly spaced SNPs through genome re-sequencing of ten *B. napus* varieties, and to validate the use of these SNPs on high-throughput genotyping platforms.

## Results

### Resequencing, SNP calling and SNP verification

Samples for resequencing were chosen from *B. napus* accessions which were parents of reference mapping populations or elite cultivars. After removing low quality and contaminant sequences, a total of 1600 million (M) paired-end reads of 75-bp or 100-bp read length (about 126 Gb total) were retained. The sequencing depth for each variety averaged 10.7 ×, ranging from 5.3 × to 37.5 × depth (Table 1). All sequence reads were aligned against the reference *B. rapa*[27] and *B. oleracea* sequences using SOAP2. Prior to alignment, all reference sequences were masked for repetitive elements using a comprehensive *Brassica* repetitive element database (unpublished). This step served to minimize the data set to low-copy DNA. SNPs were then extracted from SOAP2 alignments after a filtering scheme that (i) excluded 521 million reads with redundant hits to the reference genomes, retaining 585 million reads uniquely matched to the reference sequences; (ii) excluded 78 million SNPs supported by less than four reads in each line (at least four reads support the genotype of a single line; the SNP error rate was 1/10000); (iii) excluded 6,331,887 SNPs that were heterozygous in at least in one individual and (iv) excluded 7,224,690 SNPs with a minor allele frequency more than 0.2. After applying the filters described above, the total number of remaining SNPs was 892,803, including 892,536 bi-allelic SNPs and 267 tri-allelic SNPs. The SNP flanking sequences and the mutated sites have been deposited in NCBI dbSNP databases (ss647660101-ss657954846) [29].

**Table 1:** **Sequencing depth for ten resequenced** ***Brassica napus*** **cultivars**

Materials	Data quantity (bp)	Mean depth (×)
Zhongshuang11	41,289,270,878	37.5
73290	24,446,713,930	22.2
08-806-2	9,880,077,000	9.0
09CB01	7,875,916,400	7.2
Tapidor	7,117,473,800	6.5
XY15	7,309,111,600	6.6
09CB03	5,776,767,400	5.3
PY-2	6,615,943,400	6.0
Westar	8,343,144,400	7.6
PY-1	7,385,739,000	6.7
Total	126,040,157,808	107.9

**Figure 1:**
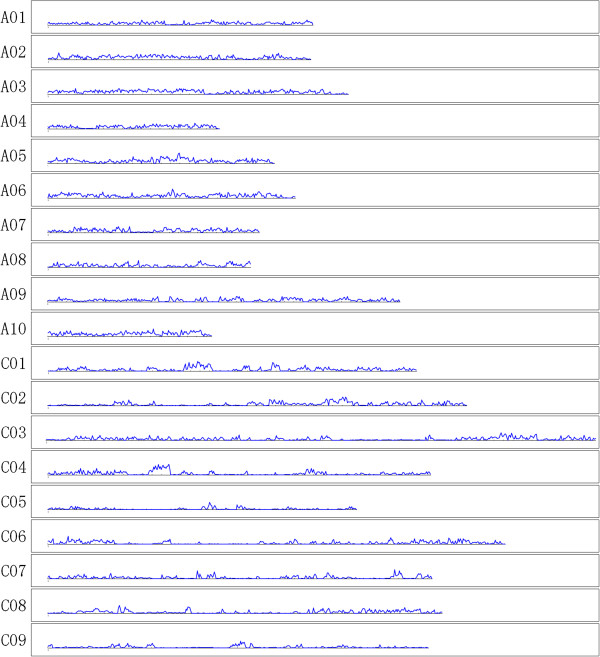
**Distribution graph for SNPs discovered in the** ***Brassica napus*** **A and C genomes.** The X axis represents the length of the chromosome while the Y axis represents the number of SNPs present at that point on each chromosome.

Transition-type SNPs comprised 57.5% of the total SNPs, while transversion-type SNPs accounted for 42.5%. The transition/transversion SNP ratio was 1.35. A total of 108,270 A/T type SNPs were detected. There were 79,533 A/T SNPs with a ‘G’ or ‘T’ base in the 3^rd^ nucleotide upstream, or a ‘C’ or ‘A’ base in the 3^rd^ nucleotide downstream: these SNPs can be efficiently genotyped using the allele-specific PCR method [30]. The largest number of SNPs were identified between PY-1 and 73290 (385,432 SNPs, 43.2%), while the smallest number of SNPs were identified between XY15 and 09CB01 (30,950 SNPs, 3.5%) (Table 2). On average, there were 210,516 SNPs between any two of the ten accessions. A total of 758,454 SNPs were distributed on the pseudochromosomes of *Brassica rapa* and *Brassica oleracea*. On average, 119 SNPs were found per 100 kb in the A genome, and 89 SNPs were found per 100 kb in the C genome. SNP frequency in the A genome was about 1.3-fold SNP frequency in the C genome (Table 3). Figure 1 shows the distribution of SNPs in the A and C genomes.

**Table 2:** **Number of SNPs detected between pairs of resequenced** ***Brassica napus*** **accessions**

	Zhongshuang11	73290	08-806-2	09CB01	Tapidor	XY15	09CB03	PY-2	Westar
**73290**	319,796								
**08-806-2**	156,255	160,437							
**09CB01**	249,524	269,446	97,961						
**Tapidor**	344,744	270,812	124,744	177,156					
**XY15**	258,030	282,982	104,584	30,950	180,195				
**09CB03**	382,962	331,329	147,992	226,364	206,498	238,157			
**PY-2**	171,536	201,921	84,905	112,203	164,533	120,094	196,957		
**Westar**	326,039	298,382	81,868	192,659	198,336	206,164	250,136	180,579	
**PY-1**	281,047	385,432	126,870	170,614	266,468	171,196	324,297	106,790	293,281

**Table 3:** **SNP distribution by chromosome for SNPs detected through resequencing of ten** ***Brassica napus*** **accessions**

Chromosome	Length	SNP	SNP/100 kb	cM	cM/100 kb
A01	24,498,464	35,077	143	82.9	0.34
A02	24,079,606	46,736	194	130.7	0.54
A03	32,789,773	58,046	177	134.1	0.41
A04	20,878,981	29,458	141	111.0	0.53
A05	23,750,921	48,510	204	142.6	0.60
A06	26,861,533	47,409	176	186.4	0.69
A07	23,303,709	35,591	153	86.6	0.37
A08	19,692,993	30,693	156	71.5	0.36
A09	35,083,316	47,753	136	188.5	0.54
A10	19,419,491	37,475	193	99.9	0.51
C01	38,761,736	47,790	123	99.5	0.26
C02	44,046,019	48,405	110	158.5	0.36
C03	57,781,479	54,055	94	161.8	0.28
C04	40,895,491	42,218	103	127.4	0.31
C05	32,828,344	14,756	45	134.6	0.41
C06	48,346,224	41,742	86	101.6	0.21
C07	40,704,487	32,438	80	133.5	0.33
C08	41,516,080	37,983	91	137.0	0.33
C09	40,126,872	22,359	56	126.3	0.31
Total	635,365,519	758,494	119	2414.4	0.38

To test empirically the quality of the SNPs identified between the ten *B. napus* accessions, ten random loci containing SNPs were validated by sequencing: PCR primers were designed from reference sequences and used to amplify DNA fragments from the ten *B. napus* accessions. Of 100 high-quality reads that aligned to reference sequence, 93 contained SNPs that matched the predicted results. Hence, the predicted false positive rate of SNP discovery was 7%.

### Non-synonymous SNP identification and enrichment analysis

**Figure 2:**
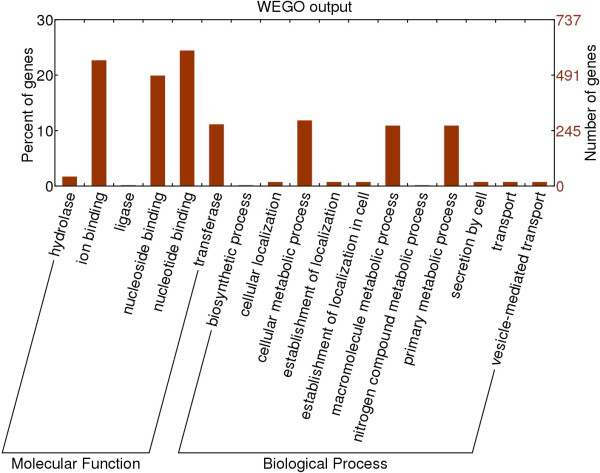
**Percentage representation of GO mappings for enriched gene categories in non-synonymous SNP-mutation-containing genes in** ***Brassica napus*** **.**

Non-synonymous SNPs that lead to an amino acid change in the protein product are of major interest. Non-synonymous variations are more likely to lead to functional mutations (‘drivers’) which may further affect phenotype. A total of 36,458 non-synonymous SNPs were identified and were located in 13,552 predicted genes. A total of 479 non-synonymous SNPs transformed stop codons to amino acid codons, whereas 505 non-synonymous SNPs transformed amino acid codons to stop codons. GO enrichment analysis predicted that the genes containing non-synonymous SNPs were involved in binding and catalytic activity more often than predicted by chance (p = 2.52E-13, Figure 2).

### High-throughput genotyping testing via the GoldenGate Array

A total of 110 candidate SNPs were submitted to Illumina Inc. to evaluate the designability of these SNPs. The rank score ranged from 0.56 to 0.99, with an average of 0.89. Fourteen SNPs with rank score 0.85 or lower were excluded, and the remaining SNPs were included in the OPA (oligonucleotide pool assay). The 96-plex OPA included 42 A-genome SNPs and 54 C-genome SNPs that were evenly distributed genome-wide.

Two mapping populations (DH and F_2_) comprising a total of 130 *B. napus* lines were genotyped using the GoldenGate assay. Clustering of Cy3 and Cy5 normalized intensities in a polar coordinate plot was used to infer the SNP genotypes. Genotype calls for all SNPs could be grouped into one or more groups depending on whether a SNP site was monomorphic or polymorphic. There were 49 SNPs which were polymorphic in at least one population. In the DH population, there were 32 polymorphic SNPs between the two parents. Six of these 32 SNPs segregated in a 1:1 ratio (p > 0.05, *χ*^2^ test). In the F_2_ population, there were 44 polymorphic SNPs between the two parents. Twelve of these 44 SNPs segregated in a 1:2:1 ratio (p > 0.05, *χ*^2^ test).

**Figure 3:**
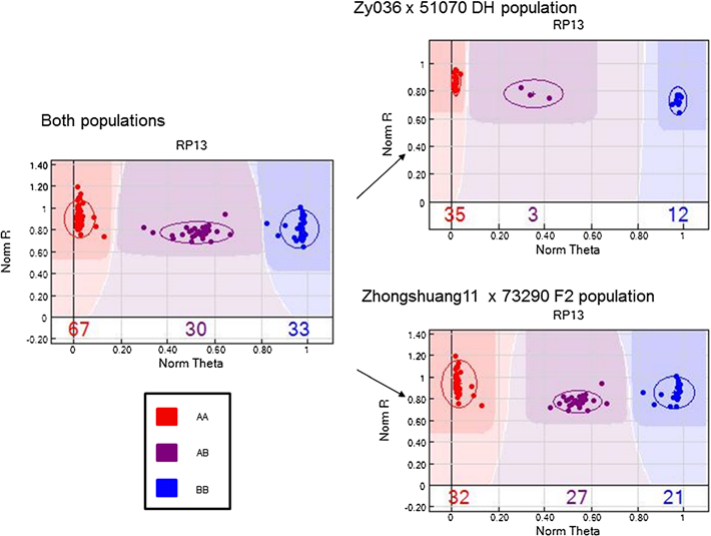
**Example of cluster compression with the GoldenGate assay, showing SNP RP13 used for genotyping the ‘ZY036’ × ‘51070’ and ‘Zhongshuang11’ × ‘73290’ populations.** The normalized R (y axis) is the normalized sum of intensities of the two channels (Cy3 and Cy5) and normalized theta (x-axis) is ((2/π)Tan-1 (Cy5/Cy3)) where a normalized theta value nearest 0 is a homozygote for allele A and a theta value nearest 1 is homozygous for allele B [42].

**Figure 4:**
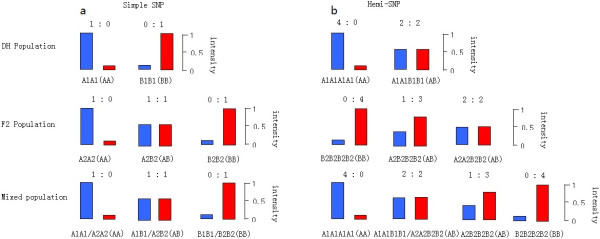
**Differences in distribution of fluorescence intensity between simple SNP and hemi-SNP.** The green bar represents the fluorescence intensity of fluorophore Cy3, while the red bar represents the fluorescence intensity of fluorophore Cy5. The genotypes of the polymorphic sites are shown in parentheses. **(a)** The distribution of fluorescence intensity for a simple SNP. The theta value could be clustered into three categories in the mixed population. **(b)** The distribution of fluorescence intensity for a hemi SNP. The theta value could be clustered into two and three categories in the individual population, while the theta value could be clustered into four or more categories in the mixed population.

In order to evaluate the reproducibility of the SNPs, three repeats of one sample were conducted for all SNP assays. Of the 96 SNPs, only one SNP showed variable results across the three repetitions. The average call rate was 92% for the 130 *B. napus* samples. In order to evaluate the reliability of the developed SNPs, the two populations were mixed and clustered with the GenomeStudio Data Analysis Software. Most SNPs were still clustered into three groups (Figure 3). There were only 3 SNPs which were clustered into four groups (Figure 4).

## Discussion

Large polyploid genomes such as *B. napus* and wheat present a challenge for SNP discovery because of the presence of multiple homoeologous sequences [25, 31]. Allelic variants need to be distinguished from non-allelic (paralog) variants (nucleotide polymorphisms between paralogs/homoeologs or between the A and C genomes) which present as false SNPs. In addition, the repetitive nature of the polyploid genomes has been one of the major obstacles to SNP discovery. In this study, three conditions were utilized to identify putative “simple SNPs”.

Firstly, low-copy DNA regions were identified by uniquely-aligned reads that were excluded from repetitive DNA regions and mapped to only one place in the *B. rapa* and *B. oleracea* reference sequences. Sequenced reads were classified into three categories: ‘uniquely aligned’, ‘repeatedly aligned’ and ‘unaligned’. Here, the ‘repeatedly aligned’ category represents duplicated loci across the allopolyploid *B. napus* genome. The ‘unaligned’ category may be partially derived from novel sequences induced by such events as genome rearrangements or transposon activity. Hence, only the uniquely aligned single-hit reads were selected from the aligned results for further analysis. Secondly, only homozygous loci were selected for subsequent analysis in each individual. Heterozygous loci could be unambiguously attributed to polymorphism between homoeologous chromosomes rather than to allelic heterozygosity. Thirdly, only reads with depth ≥ 4 were used for SNP discovery, in order to exclude SNPs generated by sequencing error. Generally speaking, minimum recommended read depth is ≥ 3 per genotype [8].

A total of 892,803 SNP polymorphisms were identified among the ten accessions of *B. napus*, using a stringent filtering approach favouring high quality SNPs over exhaustive SNP sampling to provide a resource of immediate value for crop improvement. Therefore, the actual frequency of SNP polymorphisms between these accessions is likely to have been underestimated, due to the stringent filtering methods used and due to exclusion of duplicated DNA.

In the present study, approximately 55% of SNPs were distributed on the A genome, and 45% of SNPs were distributed on the C genome. However, Bancroft et al. [32] identified 15559 SNPs on the A genome and 5675 SNPs on the C genome [32]: the bias towards A-genome SNPs was far more significant than in the present study. The genetic distance between the Ningyou7 and Tapidor C genomes is likely narrow, although these two genotypes were selected on the basis of their genetic dissimilarity, contrasting trait characteristics and different cultivation ranges [25]. However, the results of the present study agree that the A genome appears more variable than the C genome. Uneven distribution of SNPs throughout the genome is common, and has also been observed in *Brassica* relative *Arabidopsis thaliana*[33].

A total of 36,458 SNPs predicted to cause non-synonymous amino acid substitutions were identified in this study. These SNPs may represent causal genetic variation contributing to phenotype variation. Using this SNP set to perform genome-wide association in *B. napus* would be more efficient than using a general SNP set to identify causal gene mutations. GO analysis in the present study suggested that the genes predicted to contain non-synonymous SNPs were more commonly associated with binding and catalytic activity than with other functionality. This may suggest that proteins with the function of binding and catalytic activity may play a significant role in adaptive evolution.

A 96-SNP GoldenGate assay can be used successfully for SNP genotyping in *B. napus*, despite the high number of paralogous sequences in this polyploid species. Figure 3 shows an example of a putative simple SNP (SNP RP13) in the two mapping populations. If the SNP was a hemi-SNP with one homozygous locus, the genotyped samples of mixed populations would cluster into four or more groups [34] (Figure 4). In the present study, when the results from the two populations were pooled for SNP chip analysis, only 3/96 (3%) of SNPs showed four genotype clusters, suggesting these were hemi-SNPs with genotype-specific heterozygosity in the additional amplified region. However, validating these SNPs over a wider range of accessions would be valuable in determining what proportion of SNPs are simple SNPs, and what proportion are hemi-SNPs.

Although 892,803 SNPs have been developed, there are still some limitations to this work. Ten accessions was a productive number for the SNP discovery. However, increasing the number of resequenced accessions will enhance efficient, polymorphic SNP discovery. As well, eight of the accessions used were semi-winter-type *B. napus*, and therefore the effect of these SNPs in spring-type and winter-type *B. napus* needs to be further validated. Ten SNPs were randomly selected for sequencing and validation in ten lines: 97% of the sequenced SNP loci matched the prediction. The high validation ratio may have resulted from the stringent filtering conditions. Ninety-six SNPs were tested on a genotyping platform, and most polymorphic SNPs showed segregation distortion. The segregation distortion may have resulted from selection bias for particular alleles during the process of population construction (e.g. microspore culture to produce the DH population). It is also possible that for some of these SNPs genotyping using the GoldenGate assay resulted in theincorrect grouping of multiple genotype clusters together (e.g. AAAB and AABB), which would result in distorted segregation ratios. However, multi-locus genotypes are usually clearly identifiable in Genome Studio by the presence of additional separate clusters, so it is more likely that the segregation distortion observed was due to selective pressure for one or the other parental allele under population growth conditions. Future work could include validation of the genomic location of these SNPs by designing and using arrays in large mapping populations originating from diverse *B. napus* parent genotypes.

## Conclusions

A total of 892,536 bi-allelic SNP markers were developed for allopolyploid *B. napus*. The average number of SNPs per 100 kb was 119 and 89 in the A genome and C genome respectively. Transition-type SNPs accounted for 57.5% of all SNPs, and transversion-type SNPs accounted for 42.5%. A subset of developed SNPs was tested through sequencing of PCR amplification products and the GoldenGate genotyping technique, and it is predicted that the majority of the SNPs identified in this study (>450,000) can be applied in the development of much larger arrays of informative SNPs, such as Infinium II assays.

## Methods

### Plant materials

A total of ten representative accessions were chosen for SNP marker development. These comprised eight semi-winter type accessions: ‘Zhongshuang11’, ‘73290’, ‘08-806-2’, ‘09CB01’, ‘Xiangyou15’, ‘09CB03’, ‘PY-1’ and ‘PY-2’; one winter-type accession, ‘Tapidor’; and one spring-type accession, ‘Westar’. Two *B. napus* populations were used to validate the SNPs for high-throughput genotyping via GoldenGate Array. The first set comprised 92 lines of a DH population generated from crossing parents ‘zy036’ and ‘51070’ [35], and the second set comprised 250 lines of a RIL population generated from crossing parents ‘zhongshuang11’ and ‘73290’. A total of 50 lines from the DH population and 80 lines from the F_2_ population were genotyped using the GoldenGate Array.

### Genomic DNA preparation and sequencing

Seeds of ten *B. napus* accessions were germinated at 25°C on MS medium in a dark chamber. After five days, etiolated seedlings were collected for genomic DNA extraction using a standard CTAB (cetyl trimethylammonium bromide) protocol [36]. Sequencing libraries were constructed according to the manufacturers’ instructions (Illumina). Short reads were generated by applying the base-calling pipeline Solexa Pipeline-0.3 (Illumina). The Illumina sequence data have been deposited in the NCBI Sequence Read Archive (GenBank: SRA057227).

### Sequence analysis, SNP detection and verification

The *Brassica rapa* (*Brassica rapa* Release v1.01) [27] and *Brassica oleracea* assembled scaffolds (unpublished) were combined and repeat sequences were masked using RepeatMasker software. Short Oligonucleotide Alignment Program 2 (SOAP2) [37] was used to map raw pair-end reads on to reference sequences with suitable parameters for sequence similarity (−v 5), seed size (−l 32) and minimal alignment length (−s 40). On the basis of the mapping results, reads were classified into three categories: ‘uniquely aligned’, ‘repeatedly aligned’ and ‘unaligned’. SNPs detection comprised four consecutive steps. Firstly, ‘uniquely aligned’ reads (only one hit to the reference genome sequences) were selected, in order to avoid paralogue interference. Secondly, genotype data which had more than four reads of each line mapped to the reference genome was extracted. Thirdly, any alleles heterozygous within an accession were eliminated. Fourthly, SNPs where the less common allele was present in at least two accessions were selected. Custom perl scripts were used to perform these steps according to the published documents [8, 25]. Finally, 10 SNPs were randomly selected throughout the genome and SNP-flanking PCR primers (Table 4) were designed using Primer3plus [38] for fragment amplification. Primers fulfilled design parameters of 18–22 bp length, 50%–60% GC content, and 58–62°C Tm. Genomic DNA (50 ng) was used as the PCR template for amplification with the following reagents and conditions: 1× Pfu buffer, 0.2 mM dNTP mix, 1 μM primer mix, 2.5 units of Taq, and 0.2 mU of Pfu for 35 cycles under the appropriate annealing temperatures using a DNA Engine Peltier Thermal Cycler. The amplified products were sequenced using an ABI3730 sequencer by BGI.

**Table 4:** **Primers used for sequencing validation of SNPs discovered between ten** ***Brassica napus*** **accessions**

Primer_Name	SNP_type	Locus	Forward_primer	Tm(°C)	Reverse primer	Tm(°C)
ns001	A/G	BRscaffold000003-1385327	CATCAGGGAAATGGAGAGGA	60	GTGCACCAGCTCTCAAACAA	60
ns002	A/G	BRscaffold000027-2243593	CGGTTTAGGATCCGAGTTGA	60	CACGTCGCTACTGCAGCTTA	60
ns003	A/G	BOscaffold000050-1147091	CAGTGCTTGGCTCGTGTCTA	60	ATTCTGAATTCCGTTGACCG	60
ns004	G/T	BRscaffold000039-1828478	TCTGTCGGCTCTGTCATCTG	60	TCCGGTTCAGTTTCTGGTTC	60
ns005	A/G	BOscaffold000131-442418	GCTTTTGGTGTGGACATCCT	60	GAGATCCTGGGTCAACCAAA	60
ns006	G/C	BOscaffold000197-783005	CGATCGTCATACTCGGACCT	60	TTCCGATTCTGCCTCCTCTA	60
ns007	G/C	BOscaffold000230-556330	GCAGCTGATATTGCTGTGGA	60	TTGTTTCAATCCGCACAAAG	60
ns008	A/G	BOscaffold000244-357760	CGTAACGTTTGGGCTGTTTT	60	ATGGTCGGCCATGTTTTTAG	60
ns009	C/T	BOscaffold000265-92497	CACTAGCTTCGCATCAACCA	60	TGAGGTGTCATCGATAAGCG	60
ns010	C/T	BRscaffold000130-113927	TGATCGGGTTGTACACATGG	60	AGGACGGCCTTCATTATTCT	58

### SNP annotation and enrichment analysis

The localization of SNPs in coding regions was based on annotation of gene models as provided by the Brassica Genome Database (http://www.ocri-genomics.org/bolbase/). Gene families were annotated using hmmer3 software [39] via the Pfam gene family database (Pfam26.0) [40]. Enrichment analysis for the supplied gene list was carried out based on the algorithm presented by GOstat [41], with the whole set of genes from *B. rapa* and *B. oleracea* as the background. All genes with non-synomyous SNPs were extracted via custom Perl script. The GO annotations of these genes were extracted from the Brassica Genome Database. The p-value was approximated by Pearson’s chi-squared test. Fisher’s exact test was used when the expected value of any count was below 5.

### High-throughput genotyping via GoldenGate Array

A total of 110 SNPs were randomly selected from the identified SNP set. SNP-containing sequences were extracted and screened with RepeatMasker software (http://www.repeatmasker.org/) using the repeat databases. Repeats in the SNP-containing sequences were replaced with lowercase letters prior to submission to Illumina Inc. to undergo a preliminary design phase of the custom oligo pool assay (OPA), which contains the allele-specific oligoes and locus-specific oligoes for all SNPs included in the assay. A designability rank score was given to each SNP by Illumina. Scores ranged from 0 to 1.0, where a rank score of <0.4 indicated a low success rate, 0.4 to 0.6 indicated a moderate success rate, and >0.6 indicated a high success rate for the conversion of a SNP into a successful GoldenGate assay. The GoldenGate assay was performed according to the manufacturer’s protocol and as described in Fan et al. [42].

Genomic DNA was extracted from leaf tissues of 130 individuals. A NanoDrop spectrophotometer was used to ascertain that DNA quality and quantity met the requirements for the genotyping assay. Genotyping was performed using the Illumina GoldenGate Assay platform [43] and the resulting data were visualized and analyzed with the GenomeStudio Data Analysis Software package (1.0.2.20706, Illumina Inc.). Samples with a call rate lower than 0.8 and loci showing poor clustering were excluded. Accepted SNPs were manually re-clustered, to correct errors in allele calling due to inappropriate cluster identification.

## References

[1] Masterson J (1994). Stomatal size in fossil plants: evidence for polyploidy in majority of angiosperms. Science.

[2] UN (1935). Genome analysis in Brassica with special reference to the experimental formation of B. napus and peculiar mode of fertilization. Jpn J Bot.

[3] Lysak MA, Koch MA, Pecinka A, Schubert I (2005). Chromosome triplication found across the tribe Brassiceae. Genome Res.

[4] Town CD, Cheung F, Maiti R, Crabtree J, Haas BJ, Wortman JR, Hine EE, Althoff R, Arbogast TS, Tallon LJ (2006). Comparative genomics of Brassica oleracea and Arabidopsis thaliana reveal gene loss, fragmentation, and dispersal after polyploidy. Plant Cell.

[5] Fredman D, White SJ, Potter S, Eichler EE, Den Dunnen JT, Brookes AJ (2004). Complex SNP-related sequence variation in segmental genome duplications. Nat Genet.

[6] Kaur S, Francki MG, Forster JW (2011). Identification, characterization and interpretation of single‒nucleotide sequence variation in allopolyploid crop species. Plant Biotechnol J.

[7] Ganal MW, Altmann T, Röder MS (2009). SNP identification in crop plants. Curr Opin Plant Biol.

[8] Santosh K, Travis WB, Sylvie C (2012). SNP Discovery through next-generation sequencing and its applications. Int J Plant Genomics.

[9] Atwell S, Huang YS, Vilhjálmsson BJ, Willems G, Horton M, Li Y, Meng D, Platt A, Tarone AM, Hu TT (2010). Genome-wide association study of 107 phenotypes in Arabidopsis thaliana inbred lines. Nature.

[10] Gupta P, Rustgi S, Mir R (2008). Array-based high-throughput DNA markers for crop improvement. Heredity.

[11] Kim S, Misra A (2007). SNP genotyping: technologies and biomedical applications. Annu Rev Biomed Eng.

[12] Subbaiyan GK, Waters DL, Katiyar SK, Sadananda AR, Vaddadi S, Henry RJ (2012). Genome-wide DNA polymorphisms in elite indica rice inbreds discovered by whole-genome sequencing. Plant Biotechnol J.

[13] Xu X, Liu X, Ge S, Jensen JD, Hu F, Li X, Dong Y, Gutenkunst RN, Fang L, Huang L (2011). Resequencing 50 accessions of cultivated and wild rice yields markers for identifying agronomically important genes. Nat Biotechnol.

[14] Bachlava E, Taylor CA, Tang S, Bowers JE, Mandel JR, Burke JM, Knapp SJ (2012). SNP discovery and development of a high-density genotyping array for sunflower. PLoS One.

[15] Hansey CN, Vaillancourt B, Sekhon RS, de Leon N, Kaeppler SM, Buell CR (2012). Maize (Zea mays L.) genome diversity as revealed by RNA-sequencing. PLoS One.

[16] Trick M, Adamski NM, Mugford SG, Jiang CC, Febrer M, Uauy C (2012). Combining SNP discovery from next-generation sequencing data with bulked segregant analysis (BSA) to fine-map genes in polyploid wheat. BMC Plant Biol.

[17] Ferguson ME, Hearne SJ, Close TJ, Wanamaker S, Moskal WA, Town CD, de Young J, Marri PR, Rabbi IY, de Villiers EP (2011). Identification, validation and high-throughput genotyping of transcribed gene SNPs in cassava. Theor Appl Genet.

[18] Cao J, Schneeberger K, Ossowski S, Gunther T, Bender S, Fitz J, Koenig D, Lanz C, Stegle O, Lippert C (2011). Whole-genome sequencing of multiple Arabidopsis thaliana populations. Nat Genet.

[19] Tian F, Bradbury PJ, Brown PJ, Hung H, Sun Q, Flint-Garcia S, Rocheford TR, McMullen MD, Holland JB, Buckler ES (2011). Genome-wide association study of leaf architecture in the maize nested association mapping population. Nat Genet.

[20] Huang X, Wei X, Sang T, Zhao Q, Feng Q, Zhao Y, Li C, Zhu C, Lu T, Zhang Z (2010). Genome-wide association studies of 14 agronomic traits in rice landraces. Nat Genet.

[21] Kump KL, Bradbury PJ, Wisser RJ, Buckler ES, Belcher AR, Oropeza-Rosas MA, Zwonitzer JC, Kresovich S, McMullen MD, Ware D (2011). Genome-wide association study of quantitative resistance to southern leaf blight in the maize nested association mapping population. Nat Genet.

[22] Durstewitz G, Polley A, Plieske J, Luerssen H, Graner E, Wieseke R, Ganal M (2010). SNP discovery by amplicon sequencing and multiplex SNP genotyping in the allopolyploid species Brassica napus. Genome.

[23] Trick M, Long Y, Meng J, Bancroft I (2009). Single nucleotide polymorphism (SNP) discovery in the polyploid Brassica napus using Solexa transcriptome sequencing. Plant Biotechnol J.

[24] Wang W, Huang S, Liu Y, Fang Z, Yang L, Hua W, Yuan S, Liu S, Sun J, Zhuang M (2012). Construction and analysis of a high-density genetic linkage map in cabbage (Brassica oleracea L. var. capitata). BMC Genomics.

[25] Bancroft I, Morgan C, Fraser F, Higgins J, Wells R, Clissold L, Baker D, Long Y, Meng J, Wang X (2011). Dissecting the genome of the polyploid crop oilseed rape by transcriptome sequencing. Nat Biotechnol.

[26] Bus A, Hecht J, Huettel B, Reinhardt R, Stich B (2012). High-throughput polymorphism detection and genotyping in Brassica napus using next-generation RAD sequencing. BMC Genomics.

[27] Wang X, Wang H, Wang J, Sun R, Wu J, Liu S, Bai Y, Mun JH, Bancroft I, Cheng F (2011). The genome of the mesopolyploid crop species Brassica rapa. Nat Genet.

[28] Cheng F, Liu S, Wu J, Fang L, Sun S, Liu B, Li P, Hua W, Wang X (2011). BRAD, the genetics and genomics database for Brassica plants. BMC Plant Biol.

[29] Sherry S, Ward MH, Kholodov M, Baker J, Phan L, Smigielski E, Sirotkin K (2001). dbSNP: the NCBI database of genetic variation. Nucleic Acids Res.

[30] Liu J, Huang S, Sun M, Liu S, Liu Y, Wang W, Zhang X, Wang H, Hua W (2012). An improved allele-specific PCR primer design method for SNP marker analysis and its application. Plant Methods.

[31] Lai K, Duran C, Berkman PJ, Lorenc MT, Stiller J, Manoli S, Hayden MJ, Forrest KL, Fleury D, Baumann U (2012). Single nucleotide polymorphism discovery from wheat next-generation sequence data. Plant Biotechnol J.

[32] Qiu D, Morgan C, Shi J, Long Y, Liu J, Li R, Zhuang X, Wang Y, Tan X, Dietrich E (2006). A comparative linkage map of oilseed rape and its use for QTL analysis of seed oil and erucic acid content. Theor Appl Genet.

[33] Feltus FA, Wan J, Schulze SR, Estill JC, Jiang N, Paterson AH (2004). An SNP resource for rice genetics and breeding based on subspecies indica and japonica genome alignments. Genome Res.

[34] Hyten DL, Song Q, Choi IY, Yoon MS, Specht JE, Matukumalli LK, Nelson RL, Shoemaker RC, Young ND, Cregan PB (2008). High-throughput genotyping with the GoldenGate assay in the complex genome of soybean. Theor Appl Genet.

[35] Sun M, Hua W, Liu J, Huang S, Wang X, Liu G, Wang H (2012). Design of new genome-and gene-sourced primers and identification of QTL for seed oil content in a specially high-oil brassica napus cultivar. PLoS One.

[36] Doyle JJ, Doyle JL (1987). A rapid DNA isolation procedure for small quantities of fresh leaf tissue. Phytochemical Bulletin.

[37] Li R, Yu C, Li Y, Lam TW, Yiu SM, Kristiansen K, Wang J (2009). SOAP2: an improved ultrafast tool for short read alignment. Bioinformatics.

[38] Untergasser A, Nijveen H, Rao X, Bisseling T, Geurts R, Leunissen JAM (2007). Primer3Plus, an enhanced web interface to Primer3. Nucleic Acids Res.

[39] Eddy SR (2009). A new generation of homology search tools based on probabilistic inference. Genome Inform.

[40] Punta M, Coggill PC, Eberhardt RY, Mistry J, Tate J, Boursnell C, Pang N, Forslund K, Ceric G, Clements J (2012). The Pfam protein families database. Nucleic Acids Res.

[41] Beißbarth T, Speed TP (2004). GOstat: find statistically overrepresented gene ontologies within a group of genes. Bioinformatics.

[42] Fan JB, Oliphant A, Shen R, Kermani B, Garcia F, Gunderson K, Hansen M, Steemers F, Butler S, Deloukas P (2003). Highly Parallel SNP Genotyping. Cold Spring Harbor Symposia on Quantitative Biology.

[43] Fan JB, Gunderson KL, Bibikova M, Yeakley JM, Chen J, Wickham Garcia E, Lebruska LL, Laurent M, Shen R, Barker D (2006). Illumina universal bead arrays. Methods Enzymol.

